# Family physicians and the big question: A universal income grant for South Africa?

**DOI:** 10.4102/safp.v65i1.5694

**Published:** 2023-03-22

**Authors:** Gert J.O. Marincowitz, Clara Marincowitz

**Affiliations:** 1 Department of Family Medicine, Faculty of Health Sciences, University of Limpopo, Mankweng, South Africa; 2 Department of Psychiatry, Faculty of Health Sciences, University of Stellenbosch, Tygerberg, South Africa; 3 SA Medical Research Council, Cape Town, South Africa

Recently the American Academy of Family Physicians urged its members to be informed about the impact of poverty on health and to collaborate to find solutions.^1^ The unique role of family physicians in healthcare places us in a good position to advocate for policies to mitigate the effect of poverty on health.^1^ In South Africa, we need this now more than ever. Approximately half (49.2%) of our adult population was already living below the upper-bound poverty line (UBPL) in 2015. This means that we have at least 17–18 million adults living on less than R1417.00 per month.^2^ Yet, we have an average gross domestic product (GDP) per capita of $13 526.00 per year (R243 468.00/year or R20 289.00/month).^3^ There are many countries in the world with a lower GDP per capita than South Africa, and out of 191 countries, we rank number 92.^3^

Years ago, Wilkinson and Picket argued^4^ that inequality, and not poverty alone, is the factor that drives poor health outcomes. They demonstrated that in unequal societies life expectancy, obesity, heart disease, teenage pregnancy, drug abuse, and violence are all worse. These findings correlate with what Coovadia et al. highlighted in the Lancet in 2009^5^ that in South Africa we struggle with four major epidemics: violence and accidents, tuberculosis (TB) and HIV, poor maternal and child outcomes, and non-communicable diseases (NCDs) (hypertension, diabetes etc.). To further support this argument that inequality, as opposed to poverty alone, is likely a stronger driver of poor health outcomes in South Africa, see the following paragraph that compares South Africa with its peers and neighbours.

With regard to GDP per capita, our peers include countries such as Peru, Lebanon, and Sri Lanka.^3^ However, their health outcomes are markedly better than South Africa. Their Gini coefficients, a measure of inequality, are also much lower. South Africa is ranked the most unequal country in the world with a Gini coefficient of 63, and a life expectancy of 64.8 years. In comparison, Peru has a Gini coefficient of 46 and a life expectancy of 77.4 years. Sri Lanka has a Gini coefficient of 39 with a life expectancy of 77.6 years, and Lebanon has a Gini coefficient of 31.8 and a life expectancy of 79.3 years.^6,7^

As mentioned, South Africa has an annual GDP per capita of $13 526.00. Neighbouring Zimbabwe, in comparison, has an annual GDP per capita of $2434.00 with a Gini coefficient of 50.9, however their life expectancy is only slightly lower than our own at 62.16 years. Malawi on the other hand, with an annual GDP per capita of $1259.00 and a Gini coefficient of 44.9, has a life expectancy of 65.6 years. Thus, with less than 10% of South Africa’s GDP per capita, Malawi has a better life expectancy.^3,7^

These statistics suggest that inequality exacerbates the effect of poverty on health outcomes in South Africa. The introduction of a universal basic income grant (UBIG), thus has the potential to turn this around. It will reduce inequality and with that, as shown by Wilkinson and Picket,^4^ address our four major health epidemics. A UBIG could lead to a reduction in violence, and, when alcohol and substance abuse decreases, so would TB and HIV. Reducing obesity would positively impact the NCD epidemic, and a reduction in teenage pregnancy rates would improve mother and child outcomes.

A universal monthly payment of R1400.00 will come to about R700 billion per year. Our current tax revenue is around R1200bn, with an upper tax limit of 45%.^8^ The highest taxed countries in the world are: Ivory Coast (60%), Finland (56.95%), Japan (55.97%), Denmark (55.90%), Austria (55.00%), Sweden (52.90%), Belgium (50.00%), and Slovenia (50.00%).^9^ Most of these countries have very low Gini coefficients, with Slovenia having the lowest in the world at 24.6.^6^ This means that there is still room to increase our taxes. It is also notable that Finland, Denmark, Austria, and Sweden are among the top 10 happiest countries in the world, with Finland ranked as number one.^10^ Hence, high taxes do not seem to cause unhappiness at any rate.

## Conclusion

The concept of regular cash handouts (alias UBIG) may be controversial, and while future research might give us a different picture, it is not an experiment worth conducting? A UBIG could go a long way to reducing inequality and improving the social structure and health of all South Africans. For this reason, we urge our family physicians to support the introduction of a UBIG around the UBPL of R1417.00 per month. An increase in taxes might sound like a lot to ask, but considering South Africa’s long and often brutal colonial past, it is but a small price to pay.
